# Improved recovery of cell-cycle gene expression in *Saccharomyces cerevisiae* from regulatory interactions in multiple omics data

**DOI:** 10.1186/s12864-020-6554-8

**Published:** 2020-02-13

**Authors:** Nicholas L. Panchy, John P. Lloyd, Shin-Han Shiu

**Affiliations:** 1 Genetics Graduate Program, Michigan State University, East Lansing, MI 48824 USA; 2 Present address: National Institute for Mathematical and Biological Synthesis, University of Tennessee, 1122 Volunteer Blvd., Suite 106, Knoxville, TN 37996-3410 USA; 3 Department of Human Genetics and Internal Medicine, University of Michigan, Ann Arbor, MI 48109 USA; 4 Department of Computational Mathematics, Science and Engineering, Michigan State University, East Lansing, MI 48824 USA; 5 Michigan State University, Plant Biology Laboratories, 612 Wilson Road, Room 166, East Lansing, MI 48824-1312 USA

**Keywords:** Gene expression, Gene regulation, Computational biology, Machine learning, Modeling

## Abstract

**Background:**

Gene expression is regulated by DNA-binding transcription factors (TFs). Together with their target genes, these factors and their interactions collectively form a gene regulatory network (GRN), which is responsible for producing patterns of transcription, including cyclical processes such as genome replication and cell division. However, identifying how this network regulates the timing of these patterns, including important interactions and regulatory motifs, remains a challenging task.

**Results:**

We employed four in vivo and in vitro regulatory data sets to investigate the regulatory basis of expression timing and phase-specific patterns cell-cycle expression in *Saccharomyces cerevisiae*. Specifically, we considered interactions based on direct binding between TF and target gene, indirect effects of TF deletion on gene expression, and computational inference. We found that the source of regulatory information significantly impacts the accuracy and completeness of recovering known cell-cycle expressed genes. The best approach involved combining TF-target and TF-TF interactions features from multiple datasets in a single model. In addition, TFs important to multiple phases of cell-cycle expression also have the greatest impact on individual phases. Important TFs regulating a cell-cycle phase also tend to form modules in the GRN, including two sub-modules composed entirely of unannotated cell-cycle regulators (*STE12-TEC1* and *RAP1-HAP1-MSN4*).

**Conclusion:**

Our findings illustrate the importance of integrating both multiple omics data and regulatory motifs in order to understand the significance regulatory interactions involved in timing gene expression. This integrated approached allowed us to recover both known cell-cycles interactions and the overall pattern of phase-specific expression across the cell-cycle better than any single data set. Likewise, by looking at regulatory motifs in the form of TF-TF interactions, we identified sets of TFs whose co-regulation of target genes was important for cell-cycle expression, even when regulation by individual TFs was not. Overall, this demonstrates the power of integrating multiple data sets and models of interaction in order to understand the regulatory basis of established biological processes and their associated gene regulatory networks.

## Background

Biological processes, from the replication of single cells [63] to the development of multicellular organisms [66], are dependent on spatially and temporally specific patterns of gene expression. This pattern describes the magnitude changes of expression under a defined set of circumstances, such as a particular environment [67, 75], anatomical structure [20, 62], development process [17], diurnal cycle [5, 53] or a combination of the above [67]. These complex expression patterns are, in a large part, the consequence of regulation during the initiation of transcription. Initiation of transcription primarily depends on the transcription factors (TFs) bound to *cis-*regulatory elements (CREs), along with other co-regulators, to promote or repress the recruitment of RNA-Polymerase [37, 43, 64]. While this process is influenced by other genomic features, such as the chromatin state around the promoter and CREs [7, 44, 49], TF binding plays a central role. In addition to CREs and co-regulators, TFs can interact with other TFs to cooperatively [35, 38] or competitively [49] regulate transcription. In addition, a TF can regulate the transcription of other TFs and therefore, indirectly regulate all genes bound by that TF. The sum total of TF-target gene and TF-TF interactions regulating transcription in an organism is referred to as a gene regulatory network (GRN) [45].

The connections between TFs and target genes in the GRN are central to the control of gene expression. Thus, knowledge of GRN can be used to model gene expression patterns and, conversely, gene expression pattern can be used to identify regulators of specific types of expression. CREs have been used to assign genes into broad co-expression modules in *Saccharomyces cerevisiae* [5, 72] as well as other species [20]. This approach has also been applied more narrowly, to identify enhancer regions involved in myogenesis in Drosophila [17], the regulatory basis of stress responsive or not in *Arabidopsis thaliana* [67, 75], and the control of the timing of diel expression in *Chlamydomonas* reinhardtii [53]. These studies using CREs to recover expression patterns have had mixed success: in some cases the recovered regulators can explain expression globally [67, 75] while in other it is only applicable to a subset of the studied genes [53]. This may be explained in part by the difference in the organisms and systems being studied, but there are also differences in approach, including how GRNs are defined and whether regulatory interactions are based on direct assays, indirect assays, or computational inference.

To explore the effect of GRN definition on recovering gene expression pattern, we used the cell cycle of budding yeast, *S. cerevisiae,* which both involves transcriptional regulation to control gene expression during the cell cycle expression [13, 26] and has been extensively characterized [3, 57, 63]. In particular, there are multiple data sets defining TF-target interactions in *S. cerevisiae* on a genome-wide scale [11, 32, 58, 73]. These approaches include in vivo binding assays, e.g. Chromatin Immuno-Precipitation (ChIP) [15, 25], in vitro binding assays such as protein binding microarrays (PBM) [8, 16], and comparisons of TF deletion mutants with wildtype controls [58]. In this study, we address the central question of how well existing TF-target interaction data can explain when genes are expressed during the cell cycle using machine learning algorithms for each cell cycle phase. To this end, we also investigate whether performance could be improved by including TF-TF interactions, identifying features with high feature weight (i.e. more important in the model), and by combining interactions from different datasets in a single approach. Finally, we used the most important TF-target and TF-TF interactions from our models to characterize the regulators involved in regulating expression timing and identify the roles of both known and unannotated interactions between TFs.

## Results

### Comparing TF-target interactions from multiple regulatory data sets

**Fig. 1:**
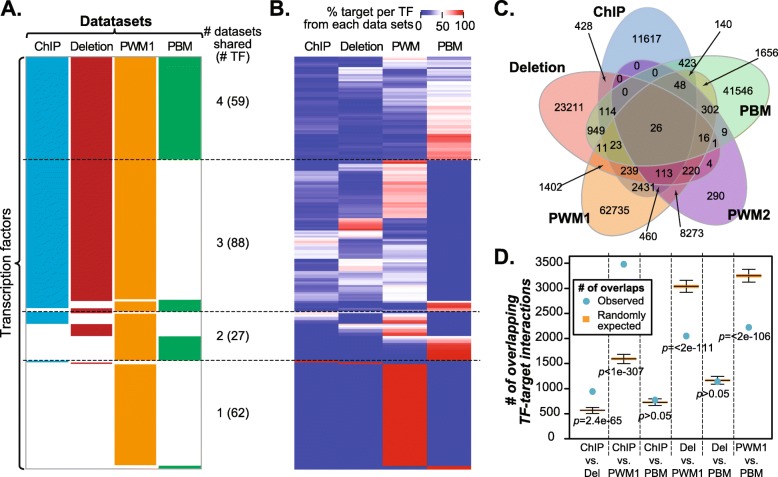
Overlap of TF and interactions between data sets. **a** The coverage of *S. cerevisiae* TFs (rows) in GRNs derived from the four data sets (columns); ChIP: Chromatin Immuno-Precipitation. Deletion: knockout mutant expression data. PBM: Protein-Binding Microarray. PWM: Position Weight Matrix. The numbers of TFs shared between datasets or that dataset-specific are indicated on the right. **b** Percentage of target genes of each *S. cerevisiae* TF (row) belonging to each GRN. Darker red indicates a higher percentage of interactions found within a data set, while darker blue indicates a lower percentage of interactions. TFs are ordered as in (**a**) to illustrate that, despite the overlap seen in (**a**), there is bias in the distribution of interactions across data sets. **c** Venn-diagram of the number of overlapping TF-target interactions from different data sets: ChIP (blue), Deletion (red), PWM1 (orange), PWM2 (purple), PBM (green). The outermost leaves indicate the number of TF-target interactions unique to each data set while the central value indicates the overlap amongst all data sets. **d** Expected and observed numbers of overlaps between TF-target interaction data sets. Boxplots of the expected number of overlapping TF-target interactions between each pair of GRNs based on randomly drawing TF-target interactions from the total pool of interactions across all data sets (see Methods). Blue filled circles indicate the observed number of overlaps between each pair of GRNs. Of these, ChIP, Deletion, and PWM1 have significantly fewer TF-target interactions with each other than expected

Although there is a single GRN which regulates transcription in an organism, different approaches to defining regulatory interactions affect how this GRN is described. Here, TF-target interactions in *S. cerevisiae* were defined based on: (1) ChIP-chip experiments (ChIP), (2) changes in expression in deletion mutants (Deletion), (3) position weight matrixes (PWM) for all TFs (PWM1), (4) a set of PWMs curated by experts (PWM2), and (5) PBM experiments (PBM; Table 1, Methods, Additional file 8: Files S1, Additional file 9: File S2, Additional file 10: File S3, Additional file 11: File S4 and Additional file 12: File S5). The number of TF-target interactions in the *S. cerevisiae* GRN ranges from 16,602 in the ChIP-chip data set to 78,095 in the PWM1 data set. This ~ 5-fold difference in the number of identified interactions is driven by differences in the average number of interactions per TF, which ranges from 105.6 in the ChIP GRN to 558.8 in the PBM GRN (Table 1). For this reason, even though most TFs were present in > 1 data sets (Fig. 1a), the number of interactions per TF is not correlated between data sets (e.g. between ChIP and Deletion, Pearson’s correlation coefficient (PCC) = 0.09; ChIP and PWM, PCC = 0.11; and Deletion and PWM, PCC = 0.046). In fact, for 80.5% for TFs, a majority of their TF-target interactions were unique to a single data set (Fig. 1b), indicating that, in spite of relatively similar coverage of TFs and their target genes, these data sets provide distinct characterizations of the *S. cerevisiae* GRN.

**Table 1:** Size and origin of GRNs defined using each data set

Data Set	TF	Target genes	# of interactions	Source
ChIP	152	4701	16,062	ScerTF
Deletion	151	5256	26,757	ScerTF
PWM1	230	6536	78,095	YeTFaSCO
PWM2	104	4740	9726	YeTFaSCO
PBM	81	4922	45,264	Zhu et al. (2009 )[73]

This lack of correlation is due to a lack of overlap of specific interactions (i.e. the same TF and target gene) between different data sets, (Fig. 1c). Of the 156,710 TF-target interactions analyzed, 89.0% were unique to a single data set, with 40.0% of unique interactions belonging to the PWM1 data set. Although the overlaps in TF-target interactions between ChIP and Deletion as well as between ChIP and PWM were significantly higher than when TF targets were chosen at random (*p* = 2.4e-65 and *p* < 1e-307, respectively, see Methods), the overlap coefficients (the size of intersection of two set divided by the size of the smaller set) were only 0.06 and 0.22, respectively. In all other cases, the overlaps were either not significant or significantly lower than random expectation (Fig. 1d). Taken together, the low degree of overlap between GRNs based on different data sets is expected to impact how models would perform. Because it remains an open question which dataset would better recover expression patterns, in subsequent sections, we explored using the five datasets individually or jointly to recover cell-cycle phase specific expression in *S. cerevisiae.*

### Recovering phase-specific expression during *S. cerevisiae* cell-cycle using TF-target interaction information

**Fig. 2:**
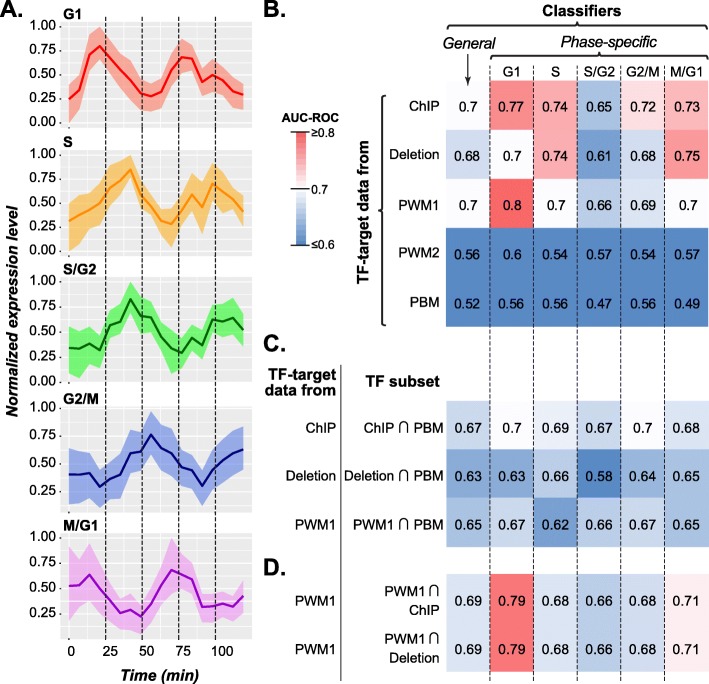
Cell-cycle phase expression and performance of classifiers using TF-interaction data. **a** Expression profiles of genes at specific phases of the cell-cycle. The normalized expression levels of gene in each phase of the cell-cycle: G1 (red), S (yellow), S/G2 (green), G2/M (blue), and M/G1 (purple). Time (x-axis) is expressed in minutes and, for the purpose of displaying relative levels of expression over time, the expression (y-axis) of each gene was normalized between 0 and 1. Each figure shows the mean expression of the phase. Horizontal dotted lines divide the timescale into 25 min segment to highlight the difference in peak times between phases. **b** AUC-ROC values of SVM classifiers for whether a gene is cycling in any cell-cycle phases (general) or in a specific phase using TFs and TF-target interactions derived from each data set. The reported AUC-ROC for each classifier is the average AUC-ROC of 100 data subsets (see Methods). Darker red shading indicates an AUC-ROC closer to one (indicating a perfect classifier) while darker blue indicates an AUC-ROC closer to 0.5 (random guessing). **c** Classifiers constructed using the TF-target interactions from the ChIP, Deletion, or PWM1 data, but only for TFs that were also present in PBM data set. Other models perform better than the PBM-based model even when restricted to the same TFs as PBM. **d** Classifiers constructed using the TF-target interactions from the PWM1 data, but only for TFs that were also present in ChIP or Deletion data set. Note that PWM1 models preform as well when restricted to TFs used by smaller data sets

Cell-cycle expressed genes were defined as genes with sinusoidal expression oscillation over the cell cycle with distinct minima and maxima and divided into five broad categories by Spellman et al. [63]. Although multiple transcriptome studies of the yeast cell cycle have been characterized since, we use the Spellman et al definition because it provides a clear distinction between the phases of the cell cycles which remains in common use [10, 12, 21, 28, 51, 54, 59, 60]. The Spellman definition of cell-cycle genes includes five phases of expression, G1, S, S/G2, G2/M, and M/G1, consisting of 71–300 genes based on the timing of peak expression that corresponds to different cell cycle phases (Fig. 2a). While it is known that each phase represents a functionally distinct period of the cell-cycle, the extent to which regulatory mechanisms are distinct or shared both within cluster and across all phase clusters has not been modeled using GRN information. Although not all of the regulatory data sets have complete coverage of cell cycle genes in *S. cerevisiae* genome, on average the coverage of genes expressed in each phase of cell-cycle was > 70% among TF-target datasets (Additional file 1: Table S1). Therefore, we used each set of regulatory interactions as features to independently recover whether or not a gene was a cell-cycle gene and, more specifically, if it was expressed during a particular cell-cycle phase. To do this, we employed a machine learning approach using a Support Vector Machine (SVM, see Methods). The performance of the SVM classifier was assessed using the Area Under Curve-Receiver Operating Characteristic (AUC-ROC), which ranges from a value of 0.5 for a random, uninformative classifier to 1.0 for a perfect classifier.

Two types of classifiers were established using TF-target interaction data. The first ‘general’ classifier sought to recover genes with cell cycle expression with at any phase. The second ‘phase specific’ classifier sought to recover genes with cell cycle expression at specific phase. Based on AUC-ROC values, both the source of TF-target interactions data (analysis of variance (AOV), *p* < 2e-16) and the phase during the cell cycle (*p <* 2e-16) significantly impact performance. Among datasets, the PBM and the expert curated PWM2 dataset have the lowest AUC-ROCs (Fig. 2b). This poor performance could be because these data sets have the fewest TFs. However, if we restrict the ChIP, Deletion and full set of PWM (PWM1) data sets to only TF present in the PBM data set, they still perform better than the PBM-based classifier (Fig. 2c). Hence, the low performance of PBM and the expert PWM must also depend on the specific interaction inferred for each TF. Conversely, if we take the full set of PWMs (PWM1), which has the most TF-target interactions, and restricts it to only include TFs present in the ChIP or Deletion datasets, performance is unchanged (Fig. 2d). Therefore, even though a severe reduction in the number of samples TF-target interactions can impact performance of our classifiers, so long as the most important TF-target interactions are covered, performance of the classifier is unaffected.

Our results indicate that both cell-cycle expression in general and timing of cell-cycle expression can be recovered using TF-target interaction data, and ChIP-based interactions alone can be used to recover all phase clusters with an AUC-ROC > 0.7, except S/G2 (Fig. 2b). Nevertheless, there remains room for improvement as our classifiers are far from perfect, particularly for expression in S/G2. One explanation for the difference in performance between phases is that S/G2 bridges the replicative phase (S) and the second growth phase (G2) of the cell-cycle that likely contains a heterogeneous set of genes with diverse functions and regulatory programs. This hypothesis is supported by the fact that S/G2 genes are not significantly over-represented in any Gene Ontology terms (see later sections). Alternatively, it is also possible that TF-target interactions are insufficient to describe the GRN controlling S/G2 expression and higher-order regulatory interactions between TFs need to be considered.

### Incorporating TF-TF interactions for recovering phase-specific expression

**Fig. 3:**
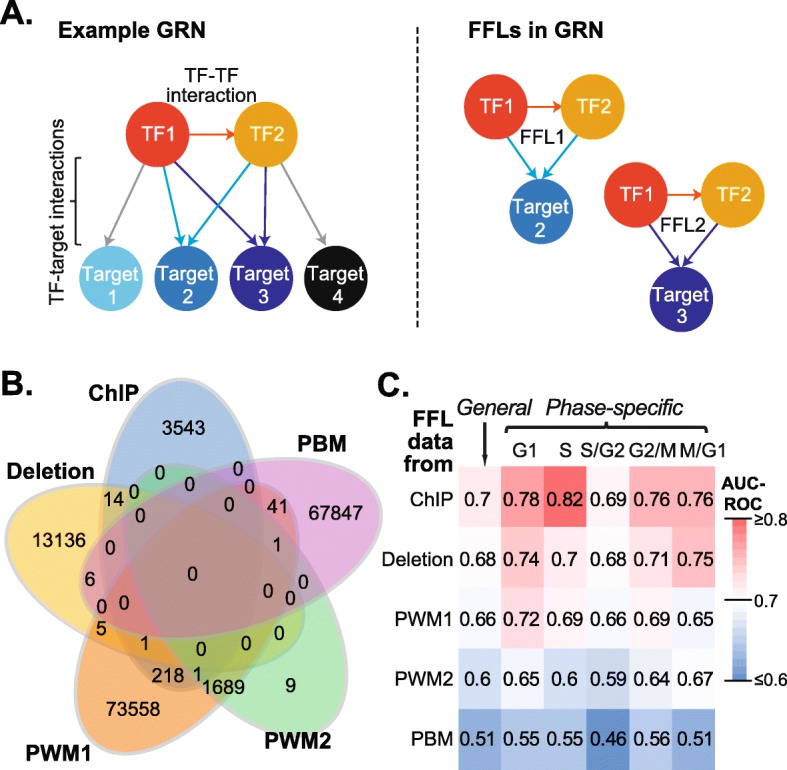
FFL definition and model performance. **a** Example Gene Regulatory Network (GRN, left) and feed-forward loops (FFLs, right). The presence of a regulatory interaction between TF1 and TF2 means that any target gene which is co-regulated by both of these TFs is part of an FFL. For example, TF1 and TF2 form an FFL with both Tar2 and Ta3, but not Tar1 or Tar4 because they are not regulated by TF2 and TF1, respectively. **b** Venn diagram showing the overlaps between FFLs identified across data sets similar to Fig. 1c. **c** AUC-ROC values for SVM classifiers of each cell-cycle expression gene set (as in Fig. 2) using TF-TF interaction information and FFLs derived from each data set. Heatmap coloring scheme is the same as that in Fig. 2b. Note the similarity and AUC-ROC value distribution here to Fig. 2b

Because a gene can be regulated by multiple TFs simultaneously, our next step was to identify TF-TF-target interactions that may be used to improve phase-specific expression recovery. Here we focused on a particular type of TF-TF interactions (i.e., a network motif), called feed forward loops (FFLs). FFLs consist of a primary TF that regulates a secondary TF and a target gene that is regulated by both the primary and secondary TF ([2]; Fig. 3a). We chose to focus on FFLs in particular because it is a simple motif involving only two regulators that is enriched in biological systems [2]. Therefore, FFLs represent a biologically significant subset of all possible two TFs interactions, which would number in the thousands even in our smallest regulatory data set. Furthermore, FFLs produce delayed, punctuated responses to stimuli, as we would expect in phase specific response, [2] and have previously been identified in cell-cycle regulation by cyclin dependent kinases [22].

We defined FFLs using the same five regulatory data sets and found that significantly more FFLs were present in each of the five GRNs than randomly expected (Table 2), indicating FFLs are an overrepresented network motif. There was little overlap between data sets ─ 97.6% of FFLs were unique to one data set and no FFL was common to all data sets (Fig. 3b). Thus, we treated FFLs from each GRN independently in machine learning. Compared to TF-target interactions, fewer cell-cycle genes were part of an FFL, ranging from 19% of all cell-cycle genes in the PWM2 dataset to 90% in PWM1 (Additional file 2: Table S2). Hence, the models made with FFLs will be relevant to only a subset of cell-cycle expressed genes. Nonetheless, we found the same overall pattern of model performance with FFLs as we did using TF-target data (Fig. 3c), indicating that FFLs were useful for identifying TF-TF interactions important for cell-cyclic expression regulation.

**Table 2:** Observed and expected numbers of FFLs in GRNs defined using different data sets

Data Set	# observed FFLs	μ expected^a^	σ^2^ expected^a^	Z-score^b^
ChIP	3777	811	28.47	104.15
Deletion	13,162	2427	49.26	217.90
PWM1	75,514	52,915	230.03	98.24
PWM2	1700	398	19.94	65.26
PBM	67,895	47,371	217.64	94.30

^a^The mean (μ) and standard deviation (σ^2^) of FFLs expected in a GRN was determined using the cube of the mean connectivity of the GRN (see Methods)

^b^The z-score reflects the difference between the observed and expected number of FFLs divided by the standard deviation of the expected number of FFLs (see Methods)

As with TF-target-based models, the best results from the FFL-based models were from GRNs derived from ChIP, Deletion, and PWM1. Notably, while the ChIP, Deletion and PWM1 TF-target-based models performed similarly over all phases (Fig. 2b), ChIP-based FFLs had the highest AUC-ROC values for all phases of expression (Fig. 3c). ChIP FFL models also had higher AUC-ROCs for each phase than those using ChIP-based TF-target interactions. However, if we used ChIP TF-target interactions to recover cell-cycle expression for the same subset of cell cycle genes covered by ChIP FFLs, the performance improves for all phases (Additional file 3: Table S3). Hence, the improved performance from using FFLs was mainly due to the subset of TFs and cell-cycle gene targets covered by the ChIP FFLs. This suggests that further improvement in cell cycle expression recovery might be achieved by including both TF-target and FFL interactions across data sets.

### Integrating multiple GRNs to improve recovery of cell-cycle expression patterns

To consider both TF-target interactions and FFLs by combining data sets, we focused on interactions identified from the ChIP and Deletion data sets because they contributed to better performance than PBM, PWM1 and PWM2 interactions (Figs. 2b, 3c**)**. We further refined our models by using subsets features (TFs for TF-Target data and TF-TF interactions for FFL data) based on their importance to the model so that our feature set would remain of a similar size to the number of cell cycle genes. The importance of these TF-target interactions and FFLs was quantified using SVM weight (see Methods) where a positive weight is correlated with cell-cycle/phase expressed genes, while a negatively weighted is correlated with non-cell-cycle/out-of-phase genes. We defined four subsets using two weight thresholds (10th and 25th percentile) with two different signs (positive and negative weights) (see Methods, Additional file 4: Table S4). This approach allowed us to assess if accurate recovery only require TF-target interactions/FFLs that include (i.e. positive weight) cell cycle genes, or if performance depends on exclusionary (i.e. negative weight) TF-target interactions/FFLs as well.

**Fig. 4:**
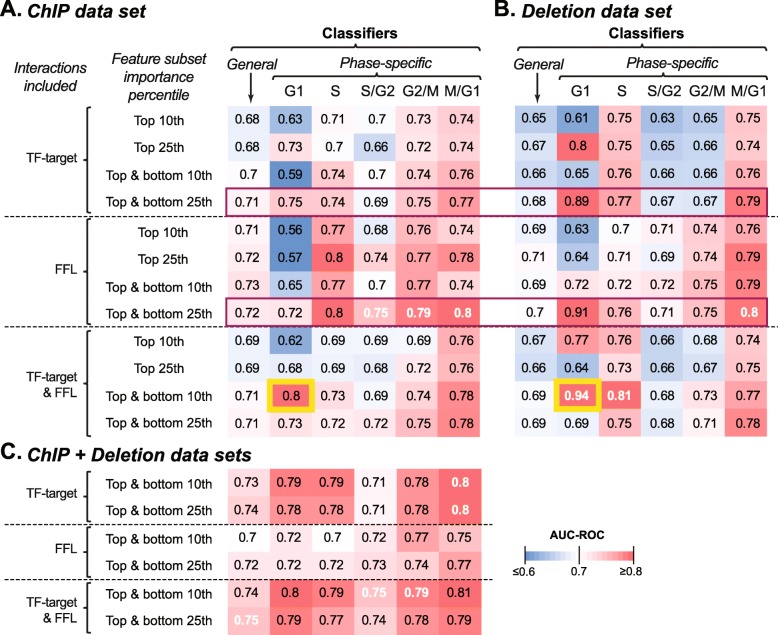
Performance of classifiers using important TF-target and/or FFL features from ChIP, Deletion, and combined data sets. **a** AUC-ROC values for models of general cycling or each phase-specific expression set constructed using a subset of ChIP TF-target interactions, FFLs, or both that had the top or bottom 10th and 25th percentile of feature weight (see Methods). The reported AUC-ROC for each classifier is the average AUC-ROC of 100 runs (see Methods). **b** As in **a** except with Deletion data. In both cases, using the 25th percentile of both features yields the best performance. **c** As in **a** except with combined ChIP-chip and Deletion data and only the top and bottom 10th and 25th subsets were used. Purple outline: highlight performance of the top and bottom 25th percentile models. Yellow outline: improved G1-specific expression recovery by combining TF-target and FFL features. White texts: highest AUC-ROC(s) for general cycling genes or genes with peak expression in a specific phase. Note that the ChIP+Deletion model have the best performance for four of the six models

First, we assessed the predictive power of cell cycle expression models using each possible subset of TF-target interactions, FFLs, and TF-target interactions/FFLs identified using ChIP (Fig. 4a) or Deletion (Fig. 4b) data. In all but one cases, models using the top and bottom 25th percentile of TF-target interactions and/or FFLs performed best when TF-target and FFL features were considered separately (purple outline, Fig. 4a, b). Combing TF-target interactions and FFLs did not always improve performance, particularly compared to FFL only models, which is to be expected given the reduce coverage of cell-cycle genes by FFL models (Additional file 3: Table S3). In contrast, if we compare TF-target only and combined models, which have similar coverage of cell cycle genes, then only M/G1 is better in TF-target only models, indicating that combing features perform better on a broader set of cell-cycles genes. Additionally, the G1 model built using the top and bottom 10th percentile of both TF-target interactions and FFLs was the best for this phase (yellow outline, Fig. 4a, b). These results suggest we can achieve equal or improved performance recovering cell-cycle by combing TF-target interactions and FFLs associated with cell-cycle (positive weight) and non-cell-cycle (negative weight) gene expression. This implies that a majority of TFs and regulatory motifs are not necessary to explain cell-cycle expression genome wide.

Next, we addressed whether combining ChIP and Deletion data improve model performance. Generally, combining these two datasets (Fig. 4c) improves or maintains model performance for the general cycling genes and most phase (white texts, Fig. 4). The ChIP+Deletion models were only outperformed by Deletion data set models for G1 and S phase. For general criteria for classifying all phases, the consistency with which classifiers built using both ChIP and Deletion data (Fig. 4c) outperformed classifiers built with just one data set (Fig. 4a, b) indicates the power of using complementary experimental data to recover expression. Additionally, these combined models outperform classifiers based on the entirety of any single data set even though they contain fewer total features. Consistent with the results of applying weight thresholds to TF-target interactions and FFLs, this suggests that only a subset of TFs and regulatory interactions need to be considered to explain the regulation of phase-specific cell cycle expression. We would expect that this subset of TFs to be enriched for known cell-cycle regulators (discussed in the next section). We also explore used this subset to determine if TFs with other functional annotation are enriched in the cell-cycle GRN and potentially identify unannotated TFs which are important to cell-cycle regulation.

### Importance and gene ontology analysis of cell-cycle regulators

**Fig. 5:**
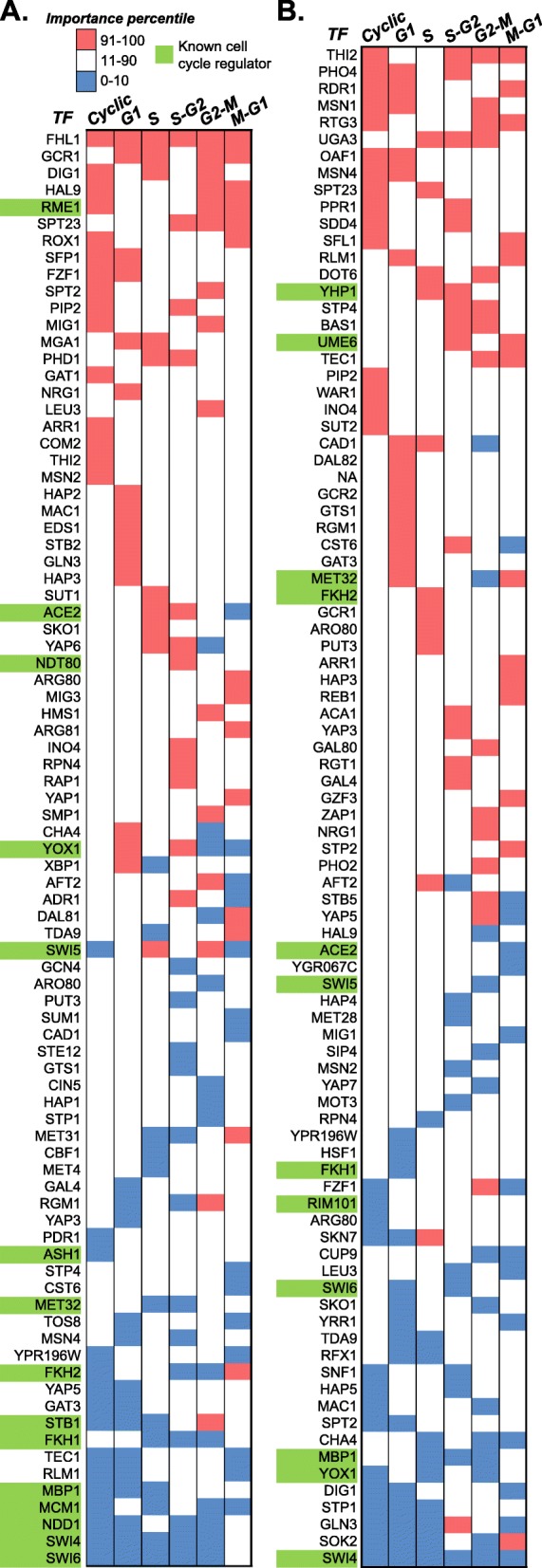
TFs with the top/bottom 10th percentile importance scores in ChIP and Deletion data-based models. Heatmap of importance of TFs in ChIP (**a**) and Deletion (**b**) data-based models. Rows represent individual TFs and columns represent models of general cycling genes (cyclic) and genes cyclic at a phase (G1, S, S-G2, G2-M, and M-G1). Blue: TFs in the lower 10th percentile of importance. Red: TFs in the upper 10th percentile of importance. White: TFs with importance between the top and bottom 10th percentiles. Green: know cell-cycle regulators. Note the frequency of TFs which are important to multiple models and the lack of correspondence between the importance of genes in ChIP and Deletion models. Additionally, there are strong correlations between the number of phases as TF is important to (weighted by whether it is positive or negative feature) and both its importance rank for the general cyclic model (*R*^2^, ChIP = 0.66, Deletion = 0.63) and the average rank across phase models (*R*^2^, ChIP = 0.84, Deletion = 0.89). As such, TF which are positively associated with many phases are have high average rank and rank in the cyclic model and vice versa, meaning the cyclic model is, in general a good predictor of the strength and sign of features

In our analysis of the ChIP and Deletion data sets, we found that performance of classifiers using only the most important TF-target interactions is similar to those using the all TF-target interactions. The top/bottom 10th percentile of TF-target interactions, which yielded the best overall performance in our final ChIP+Deletion models, include 85 TFs from the ChIP data set (Fig. 5a) and 90 TFs from the Deletion data set (Fig. 5b) are important for recovering cyclic expression in ≥1 phases. Note that TFs with the top 10th percentile importance rank are those associated with cell-cycle genes, while those in in the bottom 10th percentile importance rank are associated with non-cell cycle genes. A full listing of TFs and importance can be found in Additional file 5: Table S5. In ChIP and Deletion-based TF sets, 33 (39%) and 36 (40%) are important to > 1 phases, respectively, indicating that many cell-cycle regulators play a role in the regulation of multiple phases. However, there are only two universal regulators within each data set (SWI4 in Deletion, FHL1 in ChIP) and no universal regulator across data sets. Although 49 TF genes overlap between the ChIP and Deletion-based sets, only 9 of them are important to > 1 phases in both data sets (Fig. 5), suggesting that these two types of dataset provide unique regulatory information. Of the 25 TFs annotated as cell-cycle regulators in *S. cerevisiae* (GO:0051726), 20 and 17 were among the top 10th percentile of important features in the ChIP and Deletion data sets, respectively (green highlight, Fig. 5). Furthermore, for classifier using ChIP-chip data only, the top 10th percentile TFs are enriched for known cell-cycle regulators across all phases except M/G1 (Table 3). However, this pattern of enrichment was not found in Deletion features nor in 25th percentile of features for either data set.

**Table 3:** Enrichment *p-*values of known cell-cycle regulators among TF features important to general cell-cycle or phase-specific expression

Data Set	Top TF feature percentile	General^a^	G1^a^	S^a^	S-G2^a^	G2-M^a^	M-G1^a^
ChIP	10th	7.31e-06	0.035	0.0004	0.004	0.0007	0.085
ChIP	25th	0.0003	0.099	0.099	0.26	0.27	0.1
Deletion	10th	0.42	0.123	0.41	0.41	1	0.11
Deletion	25th	1	0.2775	1	0.78	0.27	0.58

^a^*P*-values determined by Fisher’s Exact tests

Yet, these known TFs represent a minority of TFs with high importance scores in the top 10thpercentile of TF-target interactions. To better understand the functions of these other important (i.e. large positive weight) TFs, we looked for enriched GO Terms other than cell-cycle regulation among TFs in the top 10th and 25th percentile weights in classifier for general cyclic expression using either the ChIP or the Deletion TF-target data **(**Additional file 6: Table S6). We identified 126 over-represented GO terms in total, 94 of which were unique to either ChIP-based or Deletion-based classifiers. TFs important in ChIP-based classifiers tend to be enriched in genes involved in the positive regulation of transcription in response to variety of stress conditions (e.g. freezing, genotoxicity, heat, high salinity, reactive oxygen species, and amino acid starvation; Additional file 6: Table S6). This is consistent with the finding that cell-cycle genes, particularly those involved in the G1-S phase transition, are needed for heat-shock response [34]. In contrast, TFs important to Deletion-based classifiers are enriched in categories relevant to cellular metabolism (e.g. amino acid metabolism, glycolysis, and respiration; Additional file 6: Table S6), consistent with the view that the metabolic status of the cell determines cell cycle progression [18]. The distinct functions enriched in TFs important in ChIP and Deletion data supports the hypothesis that the improvement in power from combining feature sets between ChIP and Deletion data was due to the distinct, but complementary characterization of gene regulation in *S. cerevisiae*.

### Interaction between TFs important for recovering cell-cycle expression

**Fig. 6:**
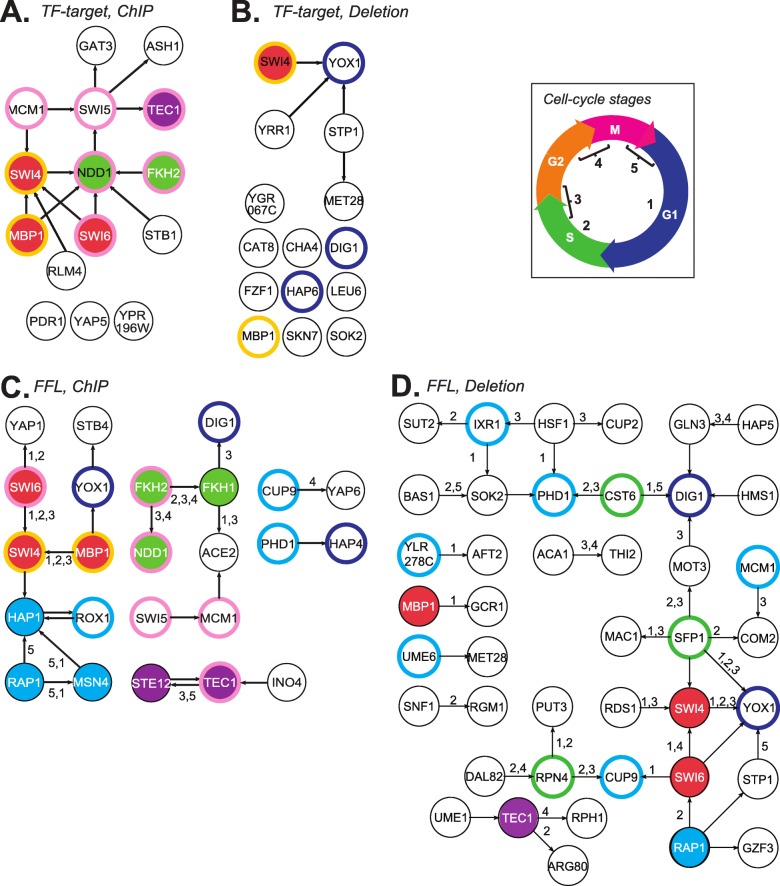
Cell-cycle GRNs based on important TF Features. (**a**, **b**) The GRNs consisting of TFs with the top 10th percentile weights for all cell-cycle expressed genes using TF-target interactions from ChIP (**a**), or Deletion (**b**) data. (**c**, **d**) The GRNs consisting of TFs in FFLs with the top 10th percentile weights for all cell-cycle expressed genes using ChIP (**c**) or deletion (**d**) data. Interactions are further annotated with the phase of cell-cycle expression they are important for (10th percentile of SVM weight in ChIP-chip models). Insert: Cell-cycle phase 1 = G1, 2 = S, 3 = S/G2, 4 = G2/M, 5 = M/G1. Red edges: new interactions identified compared to (**c**). In (**a**-**d**), node outline colors indicate TFs shared between GRNs in: orange - (**a**) and (**b**); pink - (**a**) and (**c**); blue – (**b**), (**c**), and (**d**); cyan - (**c**), and (**d**). Filled colors: four modules with TF-TF interactions important for expression in ≥2 phases. Red and green modules consist of known cell-cycle regulations, blue and purple modules consist of non-annotated cell cycle regulators

To explore the potential regulatory differences between the ChIP and Deletion datasets, we constructed ChIP and Deletion GRNs. To focus on the features with greatest importance across models, we chose the top 10th percentile of TF-target interactions from the general cell-cycle model (given the previously noted correlation between the cyclic model and importance). The resulting network shows differences in connectivity of GRNs, with only 3 of 15 TF features in the ChIP are isolated (Fig. 6a), while 10 of 15 TF are not connected to any other TF in the Deletion network (Fig. 6b). In addition, only two nodes (MBP1 and SWI4) are shared between these two GRNs (orange outline, Fig. 6a, b). This connectivity differences likely reflects the nature of the methods in assessing interactions, one direct (ChIP-chip) and the other indirect (Deletion). The *SWI6-SWI4-MBP1* module, which regulates G1/S phase transition [4, 33, 68] and part of the *FKH1-FKH2-NDD1* module, which regulates S/G 2[74] and G2/M [40] expression, are present in the ChIP but not the Deletion data-based network. We would expect this outcome for the Deletion GRN, as the 10th percentile of important TFs was not enriched for known cell cycle regulations (Table 3).

We should also point that while *SWI6-SWI4-MBP1* is present in the ChIP GRN, *FKH1* is missing (Fig. 6a), suggesting that we may be missing important interactions if we only consider TFs that are individually important. To address this issue, we also built GRNs with top 10th percentile of FFLs from general cell-cycle models based on ChIP (Fig. 6c) and Deletion (Fig. 6d) data. Since these FFLs were also used as features in phase-specific models, we labeled interactions that were above the 10th percentile of importance for individual phases (edge labels, Fig. 6c, d). In the GRN based on the ChIP FFL data (Fig. 6c), 61% interactions were important for ≥1 phases of cell-cycle expression. Furthermore, both *SWI6-SWI4-MBP1* (red) and *FKH1-FKH2-NDD1* (green) modules are fully represented in this network and are important for multiple phases of cell cycle expression (Fig. 6c). Additionally, we identified two modules that are not annotated as cell-cycle regulators in relevant GO categories. The first is the feedback loop between *STE12* and *TEC1*, which is important for both the S/G2 and M/G1 phases (purple, Fig. 6c). *STE12* and *TEC1* are known to form a complex that shares co-regulators with *SWI4* and *MBP1* to promote filamentous growth [23]. Furthermore, both genes were identified in a survey of potential cell cycle regulators which employed integrated omics data [69] and since then TEC1 has been shown to be cell cycle regulated [14]. Both TEC1 and STE12 deletions can led to cell cycle defects [19]. The second is the *RAP1-HAP1-MSN4* module, which is important for the M/G1 and G1 phases (blue, Fig. 6c). *RAP1* is involved in telomere organization [29, 42] and its association with telomeres is affected by cell cycle phases and arrest [41, 56]. *HAP1* is an oxygen response regulator [39, 65], while *MSN4* is a general stress response regulator [48, 61] and, like STE12 and TEC1, was recently shown to cause cell-cycle defects when deleted [19].

In contrast, using the 10th percentile of FFLs based the Deletion data to construct a GRN dataset revealed none of the modules uncovered using the ChIP data except *SWI4* and *SWI6* (Fig. 6d). Nonetheless, the Deletion data allows for the identification of known cell cycle regulators not found in the ChIP network, particular *SFP 1*[71] that also plays roles in regulation of ribosomes in response to stress [36, 47] (green outline, Fig. 6d). These findings highlight the importance of incorporating TF-TF interaction information, as well as both ChIP and Deletion datasets. TFs that are potentially novel cell-cycle regulators can also be identified. For example, *RPN4* regulates proteolytic stress response [46, 50, 70] and *CST6* controls carbon utilization [27](green outlines, Fig. 6d).

Overall, these findings demonstrate the utility of the FFL-based classifiers and the need to consider the importance ranks of TF-TF interaction features when recovering gene expression. The GRN constructed from carefully selected TF-TF interactions allow the recovery of regulatory modules which cannot be identified based on TF-target interaction data. Furthermore, GRNs built from the ChIP and Deletion TF-TF interactions both identified interactions important to > 1 phases of cell-cycle expression, but the characteristics of these interactions differ. ChIP-based interactions contain modules with known shared functions, while Deletion-based interactions involve central metabolism regulators like *SFP1*and consist of both direct and indirect relationships.

## Discussion

Recovering the expression of genes from their regulators and regulatory interactions remains a challenging exercise, but one that can be useful for both studying how organisms respond to various stimuli and how that response is regulated at the molecular level. Here, we have shown that the problem of recovering complex expression patterns, such as the timing of expression across the cell-cycle, directly from regulatory information can be improved using a variety of experimental and computational methods for defining gene regulatory interactions. In spite of painting distinctly different pictures of the *S. cerevisiae* GRN*,* interactions inferred from ChIP-chip, Deletion and PWM data sets were useful for characterizing genes expressed during the cell cycle and for distinguishing between cell cycle genes expressed at different phases. However, each of these data sets also has certain limitations. ChIP-chip and PBMs directly assay TF binding, but they do so outside of the context of chromatin state and other factors which regulate transcription. Deletion experiments more conclusively demonstrate that the TF affects the expression of a target gene, but do not distinguish between direct and indirect regulation. PWMs present their own challenge in that the frequency of bases may not accurately reflect actual binding site (i.e. a PWM could have a high frequency for C and G at neighboring sites, but ‘CG’ may be rare or never occur together in an actual TF binding sites). As such, the set ChIP-chip, PBM, and PWM derived interactions tend to be overly broad as only a subset of TFs with potential/proven binding at given promoter actually regulate it, while the set of Deletion TFs may be more relevant, but also, redundant because it can include TFs which indirect regulate a promoter through any already identified TF that binds it directly. It was our hope that by using a machine learning approach and integrating features, we might overcome the limitations of each individual data set to improve overall recovery.

In fact, we found that combining features from the ChIP and Deletion sets into a single model improved the overall performance and coverage of our machine learning approach, thus providing a more accurate picture of how cell-cycle timing is regulated. It is encouraging that independent models using ChIP and Deletion features both recovered a majority of annotated cell cycle TFs, but the lack of enrichment of annotated TFs and interconnectivity seen in the top Deletion features is illustrative of the limitations of using any single data set. Furthermore, using only TF-target interactions represents a significant limitation as we found that models were improved with the addition of TF-TF interactions in the form of FFLs. Particularly, a subset of the most important TF-TF interactions, combined with a subset of the most important TF-target interactions, led to models that performed better than either the full set of TF-target interactions or FFLs and allowed to identify novel regulatory interactions we would have otherwise missed.

By studying the TFs involved in the most important features of our models, we were also able to make inference about how TFs and TF-TFs interactions regulate the cell-cycle. We found many of the same TFs in the top percentile of features across models of all phases of cell cycle expression. This suggests that most TFs which are important to multiple phases of the cell cycle also have a greater impact on the phases they regulate. Therefore, these multi-phase regulators play a more central role in regulating the cell cycle compared to TFs important to only one phase. Using ChIP-chip data, we found that the top 10th percentile of important TFs from every phase except M/G1 were enriched for TFs with known cell-cycle annotations. Finally, we identified important TF-TF interactions that involve non-annotated cell-cycle regulators, such as the regulatory modules *STE12*-*TEC1* and *RAP1-MSN4-HAP1*. The *RAP1-MSN4-HAP1* module in particular stands out in that, while these regulators are individually not well correlated with cell-cycle expression, interactions between these TFs are among the most important features to recovering both cell-cycle expression in general and of the M/G1 and G1 phases in particular. Furthermore, while there was prior indication that these genes functioned during the cell cycle [19, 41, 56], unlike STE12-TEC1, there was no prior indication that *RAP1-MSN4-HAP1* might play a role in the regulation of phase specific gene expression.

Our GO analysis found that important TFs were enriched for genes associated with metabolism (*CST6*), invasive growth (*STE12*-*TEC1*), and stress responses (*RPN4*, *RAP1-MSN4-HAP1*), This was reflected in our network analysis which showed that interactions important to regulating multiple phases of cell-cycle expression were clustered around TFs involved in those processes. The identification of these unannotated regulators illustrates the importance of investigating expression regulation at the whole genome level: while there are easier ways of identifying individual cyclic genes and their potential regulators, without such a comprehensive approach the importance of these factors would be overlooked. In addition, the significance of these features is apparent only using ChIP data, further illustrating the importance of considering multiple approaches to defining GRNs.

Although our best performing model was based on data with nearly complete coverage of *S. cerevisiae* TF-DNA interactions, our models do not provide a complete picture of the regulation of cell-cycle expression. While we did include a direct assay of TF binding sites, more accurate representation of where TFs bind the promoter exist in the form of methods that incorporate information about both position and DNA modification of the binding site [22, 52]. Additionally, our approach to understanding interactions between TFs involves only FFLs, a relatively simple type of network motifs. More complicated interactions involving > 2 TFs could further improve the recovery of gene expression patterns. Nevertheless, the fact we were able to recover certain patterns of cell-cycle expression using only FFLs justifies their use in an expression modeling context. Furthermore, FFLs can be used to compose more complex interactions. For example, negative-feedback loops, which have previously been identified as being involved in the regulation of biological oscillations [9, 55], are composed of two FFL where the primary or secondary TFs are reversed. Our identification of the STE12-TEC1 interaction as important to cell-cycle expression is an example of how more complicated regulatory pathways can be captured by using their constituent FFLs.

## Conclusion

This work shows that machine learning models can provide a framework for identifying both individual regulators and multi-regulator interactions controlling temporal gene expression. Understanding the molecular basis of the timing of expression is of interest not only for the cell-cycle, but other important biological processes, such as the response to acute stresses like predation and infection and to cyclical changes in the environment including light, heat, and other cues. Although there remains room for improvement, the approach described here demonstrates that regulation of genes with time specific expression can be recovered and thus the overall methodology could potentially be applied to any expression pattern with discrete phases. The utility of this approach is further demonstrated not only by the recovery of known cell-cycle regulators and the associations between them, but also the identification of previously unannotated regulators in the form of *STE12*-*TEC1* and *RAP1-MSN4-HAP1*. Although the function of some of these genes was known to affect or be affected by the cell-cycle, our results suggests a broad, transcriptional regulatory role of phase-specific expression during the cell-cycle, which in the case of *RAP1-MSN4-HAP1* has not been sugggested before.

## Methods

### TF-target interaction data and regulatory cite mapping

Data used to infer TF-target interactions in *S. cerevisiae* were obtained from the following sources: ChIP-chip [32] and Deletion [58] data were downloaded from ScerTF (http://stormo.wustl.edu/ScerTF/), PWMs [11] and the expert curated subset of these PWMs were downloaded from YetFaSCO (http://yetfasco.ccbr.utoronto.ca/), and PBM binding scores were taken from Zhu et al. (see Supplemental Table 5, [73]). For ChIP-chip and Deletion data, the interaction between TF and their target genes were directly annotated, however, for PWMs and PBMs data we mapped inferred binding sites to the promoters of genes in *S. cerevisiae* downloaded from Yeastract (http://www.yeastract.com/). All position weight matrices were mapped for the PWM data set, however for PBM data we only used the oligonucleotides in the top 10th percentile of scores for every TF. This threshold was determined using a pilot study which found that using the 10th percentile as a cutoff maximized performance using PBM data. Mapping was done according to the pipeline previously described in Zou et al. [75] using a threshold mapping *p*-value of 1e-5 to infer a TF-target interaction.

### Overlap between TF-target interaction data

To evaluate the significance of the overlap in TF-target interactions between GRNs from different data sets, we compared the observed number of overlaps between data sets to a null distribution generated using the assumption that the association between TFs and target genes was random. Specifically, we pooled target genes from across all five data sets. Then, for each TF in each data set, selected a number of unique target genes from the pool equal to the number of interactions for that TF in the actual data set. As such, this produced a new GRN for each data set with the same number of TF-target interactions as the actual data, but with random association between TF and target genes reflective only of possible targets, not their frequency in any or all data sets. We then counted the number of overlapping features between each pair of randomized GRNs. This process was repeated 1000 times to determine the mean and standard deviation of overlap between the GRNs of each data set expected under this randomization regimen. To determine to what degree the observed overlap differed from the expectation under this random model, we evaluated the null hypothesis that the number of overlaps observed between two actual data sets is not significantly different from the null distribution produced by our randomization regime using a two-tailed z-test.

### Expected feed-forward loops in *S. cerevisiae* regulatory networks

FFLs were defined in each set of TF-target interactions as any pair of TFs with a common target genes where a TF-target interaction also existed between one TF (the primary TF) and the other (the secondary TF) which, for clarity, we refer to as a TF-TF interaction. The expected number of FFLs in each data set was determined according to the method described in “An Introduction to Systems Biology” [1]. Briefly, the expected number of FFLs (N_FFL_) in a randomly arranged GRN is approximated by the cube of the mean connectivity (λ) of the network with a standard deviation equal to the square-root of the mean. Therefore, for each data set we compared the observed number of FFLs to the expected number of FFLs from a network with the same number of connections, but with those connections randomly arranged by defining λ as the number of TF-target interactions divided by the total number of nodes (TFs + target genes) and calculating mean the standard deviation as above.

### Validating FFLs in cell-cycle expression

FFLs were validated in the context of cell-cycle expression by modeling the regulation and expression of genes involved in the FFL using a system of ordinary differential equations:

$$ \Delta \left(\begin{array}{c}S\\ {}T\end{array}\right)=\left(\begin{array}{cc}{\alpha}_S& 0\\ {}{\beta}_{S,T}& {\alpha}_T\end{array}\right)\left(\begin{array}{c}S\\ {}T\end{array}\right)+\left(\begin{array}{c}{\beta}_{P,S}\\ {}{\beta}_{P,T}\end{array}\right)f(t) $$

Where S and T are the expression of the secondary TF and target gene respectively, ∝_S_ and ∝_T_ are the decay rates of the secondary TF and target gene respectively, and β_S, T_ indicates the production rate of the target gene dependent on the secondary TF. In the nonhomogeneous term portion of the equation, β_P,S_ and β_P,T_ are the production rate of the secondary TF and target gene, respectively, which depend on the primary TF, while f(t) is the expression of the primary TF over time which is independent of both the secondary TF and the target gene. This system was solved in Maxima (http://maxima.sourceforge.net/index.html). For each FFL, maximum likelihood estimation, implemented using the bbmle package in R (https://cran.r-project.org/web/ packages/bbmle/index.html), was used to fit the model parameters to the observed expression of genes during the cell-cycle as defined by Spellman et al. [63]. Each run was initialized using the same set of initial conditions and only FFLs for which a reasonable (∝ < 0, βs > 0), non-initial parameters could be fit were kept. Between 80 and 90% of FFLs in each data set passed this threshold, while only 21% of FFLs built from random TF-TF-target triplets were fit.

### Classifying cell-cycle genes using machine learning

Recovering cell-cycle expression and the phase of cell-cycle expression was done using the Support Vector Machine (SVM) algorithm implemented in Weka [30]. We used a linear kernel so that we could later recover feature weights to evaluate feature importance. Furthermore, Han and Jiang [31] suggest that linear kernel avoid overfitting problems related to large difference between samples and still performs well compared to other kernels. In preparing out data, we treated each gene as a separate sample. The features were the presence (1) or absence (0) of TF-target and/or TF-TF interactions in FFLs defined using each of five regulatory datasets (ChIP-chip, Deletion, PWM, Expert-PWM, and PBM). For the general model, two classes were defined, cyclic and non-cyclic, based on Spellmen et al. [63](see Additional file 7: Table S7). For each SVM run, the full set of positive instances (cyclic expression) and negative instances (non-cyclic expression) was used to generate 100 balanced (i.e. 1-to-1 ratio of positive to negative) training inputs to ensure that final evaluation, which is tested against the full data set, is not biased by the fact that most of the genome it not cyclically expressed under any cell-cycle phase. Genes were only used for the input of an SVM run if at least one TF-target or TF-TF interaction feature was present. In addition to the general cell-cycle model, an SVM model was established for recovering genes in each cell-cycle phase. Models were constructed as above expect that classes were defined as expression during a specific phase of the cell-cycle, again based on data from on Spellman et al. [63]. Each balanced input set was further divided for 10-fold cross validation with SVM implemented in Weka [24, 30]. Each model was optimized using a grid search of two hyper-parameters: (1) C: the margin of the separator hyper-plane, and (2) R: the ratio of negative (non-cell cycle) to positive examples (cell-cycle) in the training set. More generally, C regulates how harshly misclassified samples are penalized in training (larger C = larger penalty) at the cost of a more rigid classifier, while R controls the frequency of cell-cycle genes in the training set (large *R* = more cell-cycle genes). The tested range of values of the two hyper-parameters were: C = (0.01, 0.1, 0.5, 1, 1.5, 2.0) and *R* = (0.25, 0.5, 1, 1.5, 2, 2.5, 3, 3.5, 4). We used the -p and -distribution options in the Weka command line to generate a class prediction output file which lists class specific scores. For each pair of hyper-parameters, performance was measured by using the score values averaged across the 100 balanced input sets to calculate the AUC-ROC. For each choice of positive class and feature set, the pair of grid search hyper-parameters which maximized the average AUC-ROC was used to define the representative model and calculate the reported AUC-ROC. Because cell cycle genes have already been identified in *S. cerevisiae* and we are interested in the underlying relationship between cell cycle genes and their regulators, we do not withhold additional samples for independent testing. Rather we use these representative models as a baseline for comparison to determine which of TF and FFLs features are most important for recovering cell cycle regulation and therefore are most likely to be biologically significant.

### Evaluating the relationship between model performance, class and feature

The effect of the phase (general cell-cycle, G1, S, S/G2, G2/M or M/G1) of expression being recovered (class) and the data set (ChIP-chip, Deletion, PWM, Expert PWM or PBM) from which TF-target interactions were derived (feature) on the performance of each SVM model was evaluated using analysis of variance (ANOVA). This was done using the “aov” function in the R statistical language using the following model:

$$ S=C+D+C\ast D $$

Where “S” is the real valued AUC-ROC score of the SVM model, “C” is a categorical feature representing the positive-class set (i.e., general, G1, S, S/G2, G2/M, or M/G1), and “D” is a categorical feature representing the data set of regulations used (i.e., ChIP, Deletion, PWM1, PWM2, or PBM).

### Importance of features to recovering cell-cycle expression

To determine the most important of features in each model, we first reran each SVM model using the best pair of parameters with the -k command line option in Weka to generate a full statistical output file which lists feature weights. Custom python scripts were then used to extract and order the weight values of the feature to define an importance rank, such that the feature with the largest positive value (most strongly associated with the positive class) had the first/highest rank and the feature with the largest negative value (most strongly associated with the negative class) had the last/lowest rank. Because multiple features often had the same weight value, we defined cutoff scores for the 10th and 25th percentile conservatively, such that the cutoff for the X^th^ percentile of positive features was smallest weight above which includes X% or less of all features and the X^th^ percentile of negative features was the largest weight below which includes X% or less of all features. The effect of this is observed most prominently in the 25th percentile features sets as ties between feature weights were more common towards the middle of the weight distributions.

### GO analysis

GO annotation for genes in *S. cerevisiae* were obtained from the Saccharomyces Genome Database (2017-1-14 version, https://downloads.yeastgenome.org/curation/literature/). The significance of enrichment of a particular term in a set of important TF compared to the incidence of the GO annotation across the genome was determined using the Fisher’s Exact Test and adjusted for multiple-hypothesis testing using the Benjamini-Hochberg method [6]. The Fisher Exact Test and multiple-hypothesis testing were implemented using the R functions fisher.test and p.adjust, respectively.

## Supplementary information

**Additional file 1: TableS1.** TF-target feature counts and target gene coverage by data set.

**Additional file 2: TableS2.** FFL feature counts and target gene coverage by data set.

**Additional file 3: Table S3.** AUR-ROC of ChIP TF-target interaction models on genes covered by ChIP FFLs.

**Additional file 4: Table S4.** Number of features in combined ChIP and Deletion models.

**Additional file 5: TableS5.** Importance of all TFs to the recovery of different phases of expression in ChIP and Deletion data sets.

**Additional file 6: TableS6.** Enrichment of GO terms in TFs important to phase of cell-cycle expression in ChIP.

**Additional file 7: Table S7.** Cell-cycle phase of *S. cerevisiae* genes.

**Additional file 8: File S1.** Lists of TF-target interactions in CHiP Data.

**Additional file 9: File S2.** Lists of TF-target interactions in Deletion data.

**Additional file 10: File S3.** Lists of TF-target interactions in All PWM data.

**Additional file 11: File S4.** Lists of TF-target interactions in Expert PWM data.

**Additional file 12: File S5.** Lists of TF-target interactions in PBM data.

## Data Availability

The dataset used in this study are available through the following means: • ChIP and TF Deletion data may be obtained from ScerTF (http://stormo.wustl.edu/ScerTF/references/) and were sourced from Harbison et al. [32] and Reimand et al. [58] respectively. • PWM for yeast TFs may be obtained from YetFaSCO (http://yetfasco.ccbr.utoronto.ca/downloads.php) • PBM data was obtained from [73] (see supplemental information, https://genome.cshlp.org/content/19/4/556.long) • *S. cerevisiae* promoter regions: Yeastract (http://www.yeastract.com/formseqretrieval.php) • *S. cerevisiae* cell cycle expression data was obtained from [63] (see supplemental information, https://www.ncbi.nlm.nih.gov/pmc/articles/PMC25624/) Additionally, TF-target interactions inferred from these data sets are available in the following Additional file 8: File S1 contains TF-target interactions from ChIP data, Additional file 9: File S2 contains TF-target interactions from Deletion data, Additional file 10: File S3 contains TF-target interactions from PWM data using all PWMs, Additional file 11: File S4 contains TF-target interactions from PWM data using only expert curated PWMs, and Additional file 12: File S5 contains TF-target interactions from PBM data.

## Abbreviations

AUC-ROC: Area under the curve of the receiver operating characteristic
ChIP: Chromatin Immuno-Precipitation
CRE: Cis-regulatory element
FFL: Feed forward loop
GRN: Gene regulatory network
PBM: Protein binding microarrays
PWM: Position weight matrix
TF: Transcription Factor

## References

[1] Alon U (2006). An introduction to systems biology: design principles of biological Circuts chapman and Hall/CRC.

[2] Alon U (2007). Network motifs: theory and experimental approaches. Nat Rev Genet.

[3] Bähler J (2005). Cell-cycle control of gene expression in budding and fission yeast. Annu Rev Genet.

[4] Bean JM, Siggia ED, Cross FR (2005). High functional overlap between MluI cell-cycle box binding factor and Swi4/6 cell-cycle box binding factor in the G1/S transcriptional program in Saccharomyces cerevisiae. Genetics.

[5] Beer MA, Tavazoie S (2004). Predicting gene expression from sequence. Cell.

[6] Benjamini Y, Hochberg Y (1995). Controlling the false discovery rate: a practical and powerful approach to multiple testing. J R Stat Soc Ser B Stat Methodol.

[7] Benveniste D, Sonntag H-J, Sanguinetti G, Sproul D (2014). Transcription factor binding predicts histone modifications in human cell lines. Proc Natl Acad Sci U S A.

[8] Berger MF, Bulyk ML (2009). Universal protein-binding microarrays for the comprehensive characterization of the DNA-binding specificities of transcription factors. Nat Protoc.

[9] Bertoli C, Skotheim JM, de Bruin RAM (2013). Control of cell cycle transcription during G1 and S phases. Nat Rev Mol Cell Biol.

[10] Blank HM, Perez R, He C, Maitra N, Metz R, Hill J, Lin Y, Johnson CD, Bankaitis VA, Kennedy BK, Aramayo R, Polymenis M (2017). Translational control of lipogenic enzymes in the cell cycle of synchronous, growing yeast cells. EMBO J.

[11] de Boer CG, Hughes TR (2012). YeTFaSCo: a database of evaluated yeast transcription factor sequence specificities. Nucleic Acids Res.

[12] Botstein D (2010). It’s the data!. Mol Biol Cell.

[13] Breeden LL (2003). Periodic transcription: a cycle within a cycle. Curr Biol.

[14] Brückner S, Kern S, Birke R, Saugar I, Ulrich HD, Möshc HU (2011). The TEA transcription factor Tec1 links TOR and MAPK pathways to coordinate yeast development. Genetics.

[15] Buck MJ, Lieb JD (2004). ChIP-chip: considerations for the design, analysis, and application of genome-wide chromatin immunoprecipitation experiments. Genomics.

[16] Bulyk ML (2007). Protein binding microarrays for the characterization of DNA-protein interactions. Adv Biochem Eng Biotechnol.

[17] Busser BW, Taher L, Kim Y, Tansey T, Bloom MJ, Ovcharenko I, Michelson AM (2012). A machine learning approach for identifying novel cell type-specific transcriptional regulators of myogenesis. PLoS Genet.

[18] Cai L, Tu BP (2012). Driving the cell cycle through metabolism. Annu Rev Cell Dev Biol.

[19] Campos SE, Avelar-Rivas JA, Garay E, Juárez-Reyes A, DeLuna A (2018). Genomewide mechanisms of chronological longevity by dietary restriction in budding yeast. Aging Cell.

[20] Chikina MD, Huttenhower C, Murphy CT, Troyanskaya OG (2009). Global prediction of tissue-specific gene expression and context-dependent gene networks in Caenorhabditis elegans. PLoS Comput Biol.

[21] Cho C-Y, Motta FC, Kelliher CM, Deckard A, Haase SB (2017). Reconciling conflicting models for global control of cell-cycle transcription. Cell Cycle.

[22] Csikász-Nagy A, Kapuy O, Tóth A, Pál C, Jensen LJ, Uhlmann F, Tyson JJ, Novák B (2009). Cell cycle regulation by feed-forward loops coupling transcription and phosphorylation. Mol Syst Biol.

[23] van der Felden J, Weisser S, Brückner S, Lenz P, Mösch H-U (2014). The transcription factors Tec1 and Ste12 interact with coregulators Msa1 and Msa2 to activate adhesion and multicellular development. Mol Cell Biol.

[24] Frank E, Hall M, Trigg L, Holmes G, Witten IH (2004). Data mining in bioinformatics using Weka. Bioinformatics.

[25] Furey TS (2012). ChIP-seq and beyond: new and improved methodologies to detect and characterize protein-DNA interactions. Nat Rev Genet.

[26] Futcher B (2002). Transcriptional regulatory networks and the yeast cell cycle. Curr Opin Cell Biol.

[27] Garcia-Gimeno MA, Struhl K (2000). Aca1 and Aca2, ATF/CREB activators in Saccharomyces cerevisiae, are important for carbon source utilization but not the response to stress. Mol Cell Biol.

[28] Grant GD, Brooks L, Zhang X, Mahoney JM, Martyanov V, Wood TA, Sherlock G, Cheng C, Whitfield ML (2013). Identification of cell cycle-regulated genes periodically expressed in U2OS cells and their regulation by FOXM1 and E2F transcription factors. Mol Biol Cell.

[29] Guidi M, Ruault M, Marbouty M, Loïodice I, Cournac A, Billaudeau C, Hocher A, Mozziconacci J, Koszul R, Taddei A (2015). Spatial reorganization of telomeres in long-lived quiescent cells. Genome Biol.

[30] Hall M, Frank E, Holmes G, Pfahringer B, Reutemann P, Witten IH (2009). The WEKA Data mining software: an update. SIGKDD Explorations.

[31] Han H, Jiang X (2014). Overcome support vector machine diagnosis Overfitting. Cancer Informat.

[32] Harbison CT, Gordon DB, Lee TI, Rinaldi NJ, Macisaac KD, Danford TW, Hannett NM, Tagne J-B, Reynolds DB, Yoo J, Jennings EG, Zeitlinger J, Pokholok DK, Kellis M, Rolfe PA, Takusagawa KT, Lander ES, Gifford DK, Fraenkel E, Young RA (2004). Transcriptional regulatory code of a eukaryotic genome. Nature.

[33] Iyer VR, Horak CE, Scafe CS, Botstein D, Snyder M, Brown PO (2001). Genomic binding sites of the yeast cell-cycle transcription factors SBF and MBF. Nature.

[34] Jarolim S, Ayer A, Pillay B, Gee AC, Phrakaysone A, Perrone GG, Breitenbach M, Dawes IW (2013). Saccharomyces cerevisiae genes involved in survival of heat shock. G3.

[35] Jolma A, Yin Y, Nitta KR, Dave K, Popov A, Taipale M, Enge M, Kivioja T, Morgunova E, Taipale J (2015). DNA-dependent formation of transcription factor pairs alters their binding specificity. Nature.

[36] Jorgensen P, Nishikawa JL, Breitkreutz B-J, Tyers M (2002). Systematic identification of pathways that couple cell growth and division in yeast. Science.

[37] Juven-Gershon T, Hsu J-Y, Theisen JW, Kadonaga JT (2008). The RNA polymerase II core promoter - the gateway to transcription. Curr Opin Cell Biol.

[38] Kazemian M, Pham H, Wolfe SA, Brodsky MH, Sinha S (2013). Widespread evidence of cooperative DNA binding by transcription factors in Drosophila development. Nucleic Acids Res.

[39] Keng T (1992). HAP1 and ROX1 form a regulatory pathway in the repression of HEM13 transcription in Saccharomyces cerevisiae. Mol Cell Biol.

[40] Koranda M, Schleiffer A, Endler L, Ammerer G (2000). Forkhead-like transcription factors recruit Ndd1 to the chromatin of G2/M-specific promoters. Nature.

[41] Laroche T, Martin SG, Tsai-Pflugfelder M, Gasser SM (2000). The dynamics of yeast telomeres and silencing proteins through the cell cycle. J Struct Biol.

[42] Laporte D, Courtout F, Tollis S, Sagot I (2016). Quiescent Saccharomyces cerevisiae forms telomere hyperclusters at the nuclear membrane vicinity through a multifaceted mechanism involving Esc1, the sir complex, and chromatin condensation. Mol Biol Cell.

[43] Lelli KM, Slattery M, Mann RS (2012). Disentangling the many layers of eukaryotic transcriptional regulation. Annu Rev Genet.

[44] Li M, Hada A, Sen P, Olufemi L, Hall MA, Smith BY, Forth S, McKnight JN, Patel A, Bowman GD, Bartholomew B, Wang MD. Dynamic regulation of transcription factors by nucleosome remodeling. Elife. 2015;4. 10.7554/eLife.06249. 10.7554/eLife.06249 PMC4456607 26047462

[45] Macneil LT, Walhout AJM (2011). Gene regulatory networks and the role of robustness and stochasticity in the control of gene expression. Genome Res.

[46] Mannhaupt G, Schnall R, Karpov V, Vetter I, Feldmann H (1999). Rpn4p acts as a transcription factor by binding to PACE, a nonamer box found upstream of 26S proteasomal and other genes in yeast. FEBS Lett.

[47] Marion RM, Regev A, Segal E, Barash Y, Koller D, Friedman N, O’Shea EK (2004). Sfp1 is a stress- and nutrient-sensitive regulator of ribosomal protein gene expression. Proc Natl Acad Sci U S A.

[48] Martínez-Pastor MT, Marchler G, Schüller C, Marchler-Bauer A, Ruis H, Estruch F (1996). The Saccharomyces cerevisiae zinc finger proteins Msn2p and Msn4p are required for transcriptional induction through the stress response element (STRE). EMBO J.

[49] Miller JA, Widom J (2003). Collaborative competition mechanism for gene activation in vivo. Mol Cell Biol.

[50] Ng DT, Spear ED, Walter P (2000). The unfolded protein response regulates multiple aspects of secretory and membrane protein biogenesis and endoplasmic reticulum quality control. J Cell Biol.

[51] O’Duibhir E, Lijnzaad P, Benschop JJ, Lenstra TL, van Leenen D, Groot Koerkamp MJA, Margaritis T, Brok MO, Kemmeren P, Holstege FCP (2014). Cell cycle population effects in perturbation studies. Mol Syst Biol.

[52] O’Malley RC, Huang S-SC, Song L, Lewsey MG, Bartlett A, Nery JR, Galli M, Gallavotti A, Ecker JR (2016). Cistrome and Epicistrome features shape the regulatory DNA landscape. Cell.

[53] Panchy N, Wu G, Newton L, Tsai C-H, Chen J, Benning C, Farré EM, Shiu S-H (2014). Prevalence, evolution, and cis-regulation of diel transcription in Chlamydomonas reinhardtii. G3.

[54] Pandey G, Zhang B, Chang AN, Myers CL, Zhu J, Kumar V, Schadt EE. An integrative multi-network and multi-classifier approach to predict genetic interactions. PLoS Comput Biol. 2010;6. 10.1371/journal.pcbi.1000928. 10.1371/journal.pcbi.1000928 PMC2936518 20838583

[55] Pett JP, Korenčič A, Wesener F, Kramer A, Herzel H (2016). Feedback loops of the mammalian circadian clock constitute Repressilator. PLoS Comput Biol.

[56] Platt JM, Ryvkin P, Wanat JJ, Donahue G, Ricketts MD, Barrett SP, Waters HJ, Song S, Chavez A, Abdallah KO (2013). Rap1 relocalization contributes to the chromatin-mediated gene expression profile and pace of cell senescence. Genes Dev.

[57] Price C, Nasmyth K, Schuster T (1991). A general approach to the isolation of cell cycle-regulated genes in the budding yeast, Saccharomyces cerevisiae. J Mol Biol.

[58] Reimand J, Vaquerizas JM, Todd AE, Vilo J, Luscombe NM (2010). Comprehensive reanalysis of transcription factor knockout expression data in Saccharomyces cerevisiae reveals many new targets. Nucleic Acids Res.

[59] Rueda C, Fernández MA, Barragán S, Mardia KV, Peddada SD (2016). Circular piecewise regression with applications to cell-cycle data. Biometrics.

[60] Santos A, Wernersson R, Jensen LJ (2015). Cyclebase 3.0: a multi-organism database on cell-cycle regulation and phenotypes. Nucleic Acids Res.

[61] Schmitt AP, McEntee K (1996). Msn2p, a zinc finger DNA-binding protein, is the transcriptional activator of the multistress response in Saccharomyces cerevisiae. Proc Natl Acad Sci U S A.

[62] Segal E, Raveh-Sadka T, Schroeder M, Unnerstall U, Gaul U (2008). Predicting expression patterns from regulatory sequence in Drosophila segmentation. Nature.

[63] Spellman PT, Sherlock G, Zhang MQ, Iyer VR, Anders K, Eisen MB, Brown PO, Botstein D, Futcher B (1998). Comprehensive identification of cell cycle-regulated genes of the yeast Saccharomyces cerevisiae by microarray hybridization. Mol Biol Cell.

[64] Spitz F, Furlong EEM (2012). Transcription factors: from enhancer binding to developmental control. Nat Rev Genet.

[65] Ter Linde JJM, Steensma HY (2002). A microarray-assisted screen for potential Hap1 and Rox1 target genes in Saccharomyces cerevisiae. Yeast.

[66] Tomancak P, Beaton A, Weiszmann R, Kwan E, Shu S, Lewis SE, Richards S, Ashburner M, Hartenstein V, Celniker SE, Rubin GM (2002). Systematic determination of patterns of gene expression during Drosophila embryogenesis. Genome Biol.

[67] Uygun S, Seddon AE, Azodi CB, Shiu S-H (2017). Predictive models of spatial transcriptional response to high salinity. Plant Physiol.

[68] Wittenberg C, Reed SI (2005). Cell cycle-dependent transcription in yeast: promoters, transcription factors, and transcriptomes. Oncogene.

[69] Wu WS, Li WH (2008). Systematic identification of yeast cell cycle transcription factors using multiple data sources. BMC Bioinformatics.

[70] Xie Y, Varshavsky A (2001). RPN4 is a ligand, substrate, and transcriptional regulator of the 26S proteasome: a negative feedback circuit. Proc Natl Acad Sci U S A.

[71] Xu Z, Norris D (1998). The SFP1 gene product of Saccharomyces cerevisiae regulates G2/M transitions during the mitotic cell cycle and DNA-damage response. Genetics.

[72] Yuan Y, Guo L, Shen L, Liu JS (2007). Predicting gene expression from sequence: a reexamination. PLoS Comput Biol.

[73] Zhu C, Byers KJRP, McCord RP, Shi Z, Berger MF, Newburger DE, Saulrieta K, Smith Z, Shah MV, Radhakrishnan M, Philippakis AA, Hu Y, De Masi F, Pacek M, Rolfs A, Murthy T, Labaer J, Bulyk ML (2009). High-resolution DNA-binding specificity analysis of yeast transcription factors. Genome Res.

[74] Zhu G, Spellman PT, Volpe T, Brown PO, Botstein D, Davis TN, Futcher B (2000). Two yeast forkhead genes regulate the cell cycle and pseudohyphal growth. Nature.

[75] Zou C, Sun K, Mackaluso JD, Seddon AE, Jin R, Thomashow MF, Shiu S-H (2011). Cis-regulatory code of stress-responsive transcription in Arabidopsis thaliana. Proc Natl Acad Sci U S A.

